# Body mass estimates of an exceptionally complete *Stegosaurus* (Ornithischia: Thyreophora): comparing volumetric and linear bivariate mass estimation methods

**DOI:** 10.1098/rsbl.2014.0984

**Published:** 2015-03

**Authors:** Charlotte A. Brassey, Susannah C. R. Maidment, Paul M. Barrett

**Affiliations:** 1 Department of Earth Sciences, The Natural History Museum, Cromwell Road, London SW7 5DB, UK; 2 Department of Earth Science and Engineering, Imperial College, South Kensington Campus, London SW7 2AZ, UK

**Keywords:** *Stegosaurus*, body mass, scaling equations, volumetric model, photogrammetry

## Abstract

Body mass is a key biological variable, but difficult to assess from fossils. Various techniques exist for estimating body mass from skeletal parameters, but few studies have compared outputs from different methods. Here, we apply several mass estimation methods to an exceptionally complete skeleton of the dinosaur *Stegosaurus*. Applying a volumetric convex-hulling technique to a digital model of *Stegosaurus*, we estimate a mass of 1560 kg (95% prediction interval 1082–2256 kg) for this individual. By contrast, bivariate equations based on limb dimensions predict values between 2355 and 3751 kg and require implausible amounts of soft tissue and/or high body densities. When corrected for ontogenetic scaling, however, volumetric and linear equations are brought into close agreement. Our results raise concerns regarding the application of predictive equations to extinct taxa with no living analogues in terms of overall morphology and highlight the sensitivity of bivariate predictive equations to the ontogenetic status of the specimen. We emphasize the significance of rare, complete fossil skeletons in validating widely applied mass estimation equations based on incomplete skeletal material and stress the importance of accurately determining specimen age prior to further analyses.

## 1. Introduction

In extant taxa, body mass is an indicator of fundamental ecological and physiological traits such as population density, metabolism and cost-of-transport [1]. Key evolutionary transitions in deep time, such as the origin of avian flight [2] and the adaptive radiation of mammals around the Cretaceous-Paleogene boundary [3], have been interpreted in the context of body size: thus body mass reconstruction in extinct species is of considerable interest.

Ideally, mass estimates for extinct taxa would be based upon complete specimens, but such material is rare. Consequently, many mass estimation techniques for fossil taxa rely upon measurements taken from commonly preserved skeletal elements. Recently, the sum of femoral and humeral circumferences was shown to correlate strongly with mass in extant taxa [4]. However, the applicability of this equation to fossil groups with unusual morphological features, overly robust/gracile limb elements or lying outside of the size range of extant taxa, remains to be tested. Additionally, application of bivariate predictive equations to specimens of uncertain ontogenetic status is potentially problematic given evidence of allometric scaling of limb dimensions with age among dinosaur taxa [5].

When specimens are complete, volumetric methods of mass estimation can be used [6,7]. Such methods incorporate all of the available data from the specimen and are not biased by the problem of unexpectedly robust/gracile elements, nor do they rely on regressions that are extrapolated beyond empirically based body mass data. Nevertheless, the reconstruction process involves a degree of subjectivity and sensitivity analyses are essential to quantify the effects of anatomical uncertainties [6]. Convex hulling [7] circumvents this problem by fitting ‘shrink-wrap’ convex polytopes around the three-dimensional skeleton and deriving a mass estimate based on the relationship between convex hull volume (*C*_vol_) and body mass in modern species.

Using a three-dimensional computer model of an exceptionally complete *Stegosaurus* specimen, we compare mass estimation techniques based on volumetric and traditional bivariate regressions to test if estimates generated from limb bone dimensions alone are biologically plausible for taxa with morphologies lacking close modern analogues or for specimens that have not attained full adult size.

## 2. Material and methods

The specimen is a *Stegosaurus stenops* (NHMUK (Natural History Museum, London) R36730) from the Upper Jurassic Morrison Formation near Shell, Wyoming. It is substantially complete, with all body regions represented except the left forelimb and part of the tail. NHMUK R36730 is classified as a ‘young adult’ based on histological sampling [8,9] (electronic supplementary material, S1). The specimen was digitized as disarticulated bones using photogrammetry [10] and the freely available software ‘VisualSFM’ (http://ccwu.me/vsfm) and ‘Meshlab’ (http://meshlab.sourceforge.net) (electronic supplementary material, S2).

The skeleton was posed in 3DsMax (www.autodesk.com/3dsmax) and a convex hull model produced (*C*_vol(pref)_) representing our preferred articulation of the elements based on comparative dinosaur anatomy and information from the extant phylogenetic bracket (crocodilians/birds). A sensitivity analysis quantified the effect of rearticulation on *C*_vol_. Intervertebral spacing, rib flaring and scapula position were altered to define a minimum (*C*_vol(min)_) and maximum (*C*_vol(max)_) volume pose for the skeleton (electronic supplementary material, S3). Models were subdivided into functional units: head, neck, trunk (sacrum and thorax), tail, upper arm, forearm, hand, thigh, shank and foot. The cervical series was subdivided to ensure a tight fit of the hulls around the neck. Convex hulls were fitted to functional units using the ‘convhulln’ function in matlab (www.mathworks.com) implementing the ‘qhull’ algorithm [11]. Total *C*_vol_ was calculated as the sum of segment values, and body mass estimated using the relationship between *C*_vol_ and body mass published elsewhere [7,12] (electronic supplementary material, S4). By directly converting *C*_vol_ into a mass estimate, a body density value is not explicitly assigned. However, there is an assumption that the density of the fossil species falls within the range of those species from which the predictive equation is derived, in this case modern quadrupedal mammals.

We expanded our ‘maximum’ convex hull model (*C*_vol(max)_) to match the body mass value predicted when applying a recently published scaling equation (*C*_vol(C&E)_) [4]. Dermal armour mass was determined separately and added to each volumetric mass estimate (electronic supplementary material, table S1). Mass estimates based on femoral and/or humeral circumference were calculated using the masstimate package in R [13] and raw data available from previous studies [14,15] (electronic supplementary material, S4). The potential effect of ontogenetic scaling was investigated using Developmental Mass Extrapolation (DME), whereby the mass of a ‘known’ adult individual (in this case *Stegosaurus* YPM (Peabody Museum of Natural History, Yale University) 1853 and YPM 1856) is estimated using the bivariate equation in question, and subsequently scaled isometrically on the basis of femoral length to the subadult individual [16] (electronic supplementary material, S5).

## 3. Results

**Figure 1.**
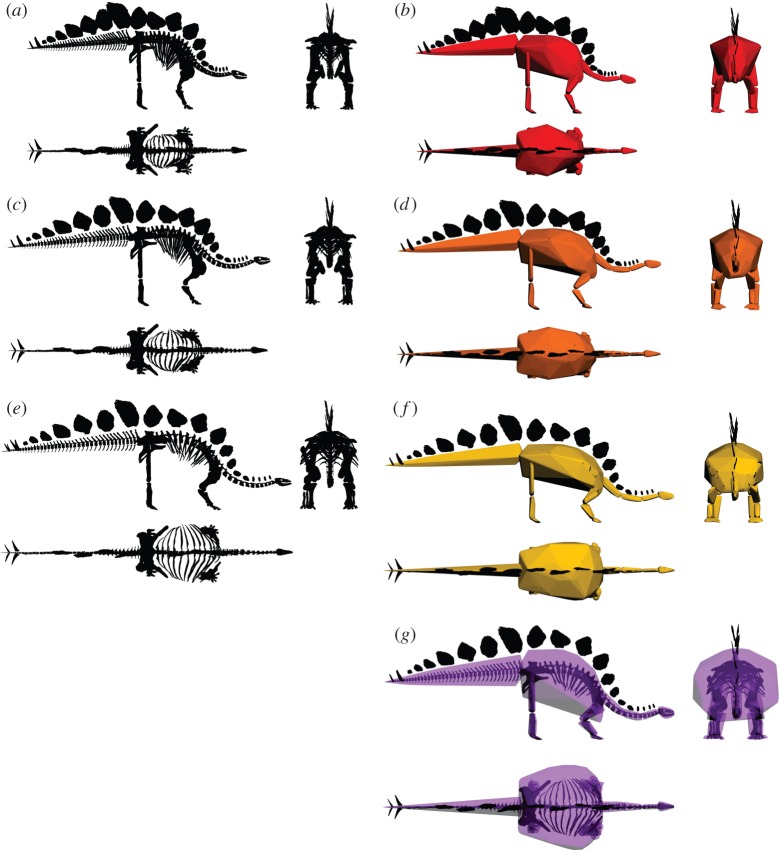
Reconstructions of *Stegosaurus stenops* (NHMUK R36730) and associated convex hulls. (*a,b*) *C*_vol(min)_; (*c,d*) *C*_vol(pref)_; (*e,f*) *C*_vol(max)_; (*g*) *C*_vol(C&E)_. (Online version in colour.)

**Figure 2.**
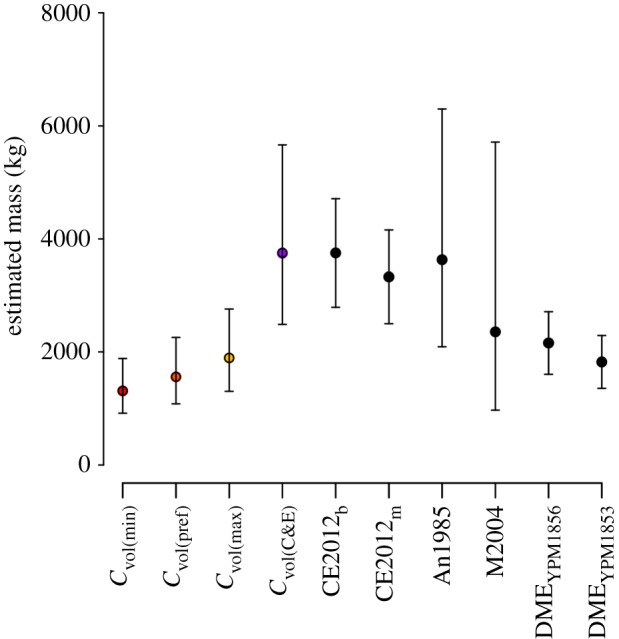
Volumetric mass estimates for NHMUK R36730 calculated here compared with those derived from proximal limb circumference. For abbreviations, see table 2. (Online version in colour.)

The convex hull reconstruction of our preferred model (*C*_vol(pref)_) provides a mass estimate of 1560 kg (95% prediction interval (PI) 1082–2256 kg), including 34 kg of dermal armour (electronic supplementary material, table S1). Values for *C*_vol(min)_ of 1311 kg (95% PI = 916–1884 kg) and *C*_vol(max)_ of 1894 kg (95% PI = 1303–2760 kg) derived from the rearticulation sensitivity analysis provide upper and lower bounds to our convex hull mass estimate (figure 1 and table 1). Prior to accounting for ontogeny, mass estimates based on proximal limb bone circumferences [5] were consistently higher than those based on convex hulls (figure 2), ranging between 2355 and 3751 kg (table 2). The prediction intervals of *C*_vol(pref)_ do not overlap those of either of the predictive equations of Campione & Evans (C&E) [4] (figure 2). To achieve the 3752 kg estimate derived from the C&E bivariate equation [4], the convex hulls fitted to the *C*_vol(max)_ model required considerable rescaling, resulting in a total convex hull volume of 3.378 m^3^ for the *C*_vol(C&E)_ model and an estimated body mass of 3745 kg (figure 1*g* and table 1). However, accounting for possible ontogenetic scaling brought volumetric and linear bivariate predictions into close agreement. Based on DME using adult *Stegosaurus* specimens (YPM1853, 1856) as endpoints, predicted masses for NHMUK R36730 using the C&E bivariate equations [4] were 1823 kg and 2158 kg, respectively, and fall within the confidence intervals of our volumetric models.

**Table 1.** Volume data (m^3^) for convex hull reconstructions of *Stegosaurus stenops* (NHMUK R36730).

body segment	*C*_vol(pref)_	*C*_vol(min)_	*C*_vol(max)_	*C*_vol(C&E)_
head	0.0056	0.0056	0.0056	0.0119
neck	0.0177	0.0152	0.0199	0.0425
trunk	1.0786	0.8724	1.3686	2.9174
left upper arm	0.0098	0.0098	0.0098	0.0208
left forearm	0.0046	0.0046	0.0046	0.0099
left hand	0.0021	0.0021	0.0021	0.0046
left thigh	0.0159	0.0159	0.0159	0.0339
left shank	0.0084	0.0084	0.0084	0.0178
left foot	0.0018	0.0018	0.0018	0.0038
right upper arm	0.0084	0.0084	0.0084	0.0180
right forearm	0.0042	0.0042	0.0042	0.0091
right hand	0.0020	0.0020	0.0020	0.0043
right thigh	0.0166	0.0166	0.0166	0.0354
right shank	0.0091	0.0091	0.0091	0.0194
right foot	0.0014	0.0014	0.0014	0.0031
tail	0.0936	0.0781	0.1061	0.2262
total	1.2800	1.0558	1.5848	3.3781

**Table 2.** Mass estimates (kg) for *Stegosaurus stenops* (NHMUK R36730) based on proximal limb circumference. ‘An1985’ and ‘M2004’ equations have been modified from those originally published (electronic supplementary material, S4). CE2012_b_ and CE2012_m_ refer to bivariate and multivariate equations in [4].

equation	mean	lower 95% PI	upper 95% PI
CE2012_b_ [4]	3752	2790	4713
CE2012_m_ [4]	3329	2499	4159
An1985 [15]	3632	2089	6316
M2004 [16]	2355	971	5717
DME_YPM1853_ [4]	1823	1356	2290
DME_YPM1856_ [4]	2158	1605	2712

## 4. Discussion

This is the first study to apply both volumetric and linear bivariate mass estimation techniques to the same *Stegosaurus* individual. Prior to accounting for ontogenetic scaling, volumetric mass estimates for *Stegosaurus* were considerably lower than those predicted on the basis of limb bone dimensions. The *C*_vol(min),_ *C*_vol(pref)_ and *C*_vol(max)_ model predictions are 35, 42 and 50%, respectively, of the 3752 kg estimated by the C&E bivariate predictive equation [4], which was based on adult individuals. Previous studies on other stegosaur specimens also found that volumetric mass estimates were consistently lower than those based on limb bone scaling (electronic supplementary material, tables S2 and S3).

The *C*_vol(C&E)_ model created to reconstruct the body dimensions required to meet the original 3752 kg mass estimation of Campione & Evans [4] represents a 165% increase in volume from the *C*_vol(pref)_ model. Outwardly, the convex hull volumes appear extremely large (figure 1*g*) and, given the excessive soft tissue volume that would need to be placed outside the skeleton in order to reach a body mass of more than 3700 kg, we consider that the CE predictive equation [5] overestimates body mass in *Stegosaurus* when applied in this manner (i.e. without correction for the ontogenetic stage of the specimen).

The convex hull mass estimates for NHMUK R36730 are lower than those calculated for other *Stegosaurus* specimens (e.g. USNM (United States National Museum of Natural History, Washington, DC, USA) 4934) using alternative volumetric techniques (electronic supplementary material, table S1). NHMUK R36730 was not osteologically mature at time of death and is smaller than USNM 4934 (electronic supplementary material, table S4). It is therefore expected that the convex hull estimates calculated here should be lower than previous volumetric reconstructions. This difference highlights the importance of generating specimen-specific mass values prior to subsequent biomechanical analyses rather than applying species-means.

Although a ‘young adult’ [8,9], NHMUK R36730 was still growing at the time of death (electronic supplementary material, S1). When DME was applied to the specimen using ‘known’ adult *Stegosaurus* as a baseline (electronic supplementary material, S5), the two mass estimation techniques converged significantly. Importantly, correction for ontogenetic stage resulted in the original mass estimates derived using the CE equations [4] falling within the confidence intervals of all volumetric estimates (figure 2 and table 2). Much of the discrepancy between the two techniques may, therefore, be attributed to the age of the specimen.

Discrepancies between volumetric and linear mass estimates have been noted for other dinosaurs [7], and our results suggest that ontogeny is a potential explanation. By considering NHMUK R36730 as subadult rather than adult, previous mass estimates [4] more than halve (from 3752 to 1823 kg; table 2). This highlights the sensitivity of linear bivariate equations to ontogenetic status and urges caution in instances when the age of the specimen is unclear (e.g. in the absence of histological information or for fragmentary material). While important palaeoecological studies of broad taxonomic scope must necessarily include as large a sample size as possible, our results suggest that authors should restrict themselves to sampling the largest individual of a given taxon in order to minimize this effect [17].

In summary, our volumetric mass estimates of *Stegosaurus* fall outside the prediction intervals of commonly used mass prediction equations based on proximal limb dimensions, a pattern seen in other dinosaurs [7]. Ontogenetic scaling is a possible explanation for this discrepancy. Rare finds of exceptionally complete specimens should play a crucial role in validating widely applied mass estimations based on incomplete skeletal material. It is possible that important size-related shifts in palaeoecology or physiology in fossil taxa are being misinterpreted owing to the inappropriate application of mass prediction equations. Our results urge caution when estimating the mass of extinct species, particularly when the ontogenetic status of the specimen is difficult to determine.

## Supplementary Material

Supplementary Material

## Data accessibility

Convex hull models are downloadable from Dryad (doi:10.5061/dryad.2307m).
