# Supplementary material for: Body mass estimates of an exceptionally complete Stegosaurus (Ornithischia: Thyreophora): comparing volumetric and linear bivariate mass estimation methods
Source: Biol Lett. 2015 Mar;11(3):20140984. doi: 10.1098/rsbl.2014.0984 (PMC4387493; doi:10.1098/rsbl.2014.0984)
Supplement: Supplementary Material [file rsbl20140984supp1.docx]

**Supplementary Material**

**Supplementary Material S1: Specimen NHMUK R36730**

This specimen of *Stegosaurus stenops* (NHMUK R36730) is one of the most complete individuals of the taxon ever found. Missing parts include some proximal caudals (~5–20), all except two chevrons, the left-hand side of the pelvis, the left forelimb, both sets of manual and some pedal phalanges. One plate is also missing. In instances where the original bones have not been preserved, material reconstructed for the mounted skeleton was scanned and included in our digital model.

A histological examination of NHMUK R36730 ([1]; listed as SMA RCR0603) found longitudinal vascularity and the absence of an external fundamental system (EFS). These histological characteristics can be used to determine the ontogenetic stage of the specimen using the histologically determined growth stages of Hayashi et al. [2]. This suggests that the specimen was growth stage 3 (‘young adult’) and indicates that while the specimen may have been sexually mature, it was still growing, a conclusion supported by the absence of an external fundamental system [1]. In addition, several skeletal features of NHMUK R36730 indicate it was not osteologically mature at time of death. Although neurocentral sutures are fully closed they are still visible on the lateral sides of the centrum, the olecranon process of the ulna is relatively small, fenestrae are present between the sacral ribs in dorsal view, the anterior trochanter of the femur is present as a distinct process, and the tarsals are not fused to the tibia and fibula.

Although the specimen is exceptionally complete, there is evidence of taphonomic alteration to the shapes of several bones. Of particular importance to the present study, both the left and right femora are anteroposteriorly crushed. As the linear bivariate equations applied here incorporate a measure of femoral circumference, this is likely to have impacted our mass estimations. Any attempt to correct femoral circumference measurements for post-mortem deformation would involve further assumptions regarding the original cross-sectional geometry of the femur. Furthermore, in many instances in the fossil record it is not clear to what extent the skeleton has undergone deformation. We have therefore used the uncorrected values for femoral circumference throughout. The right humerus is undeformed.

Some dorsal ribs appear artificially straightened, likely as a result of post-mortem crushing and subsequent consolidation during excavation. As the dorsal ribs are responsible for defining the overall geometry and volume of the trunk, it is possible that straightening of the ribs has led to a flaring-out of the ribcage (resulting in a trapezoidal, rather than ellipsoidal, cross-section for the ribcage) and an overestimation of trunk volume. However, given the paucity of well-preserved 3D *Stegosaurus* rib material, an attempt to reconstruct the geometry of the ribs would involve more uncertainties and assumptions than incorporating the uncorrected material into the 3D models. Therefore estimated trunk volume calculated here likely represents the upper bounds of trunk volume in life.

**Supplementary Material S2: Specimen digitisation**

NHMUK R36730 was digitised as disarticulated individual bones using photogrammetry prior to being mounted and placed on public display. Between ~50–200 photographs were taken of each element using a standard dSLR camera and photogrammetric models were created in VisualSFM [3]. In some instances two separate photogrammetric models corresponding to anterior/posterior surfaces (long bones) or dorsal/ventral surface (pelvic block) were created and subsequently stitched together in Meshlab [4]. In other instances, one single model was created from photographs of the element lying on its ventral surface and some hole-filling was required in Meshlab to create a closed-manifold watertight object. Metal bars forming part of the armature upon which the specimen is now mounted had previously been adhered to the cervical and dorsal ribs. Photogrammetric models were therefore created with the metal components in place, and subsequently removed where necessary in Meshlab. The plates were not digitised using photogrammetry due to their extremely thin and fragile nature. Plates were instead digitised using a Romer Absolute Arm V5 surface laser scanner and subsequently reconstructed in Polyworks software ([www.innovmetric.com](http://www.innovmetric.com)).

**Supplementary Material S3: Articulation of digital models**

We carried out a sensitivity analysis to quantify the effect of uncertainty in model rearticulation on total body volume by creating a minimum (*C*_vol(min)_) and maximum (*C*_vol(max)_) volume digital model. In order to account for an unknown thickness of articular cartilage between vertebrae, vertebrae in *C*_vol(min)_ were brought close together such that the zygapophyses overlapped to an extreme degree and the centra of adjacent vertebrae made contact. To represent the greatest possible intervertebral spacing in *C*_vol(max)_, the vertebrae were spaced apart such that the zygapophyses no longer overlapped and large spaces were visible between the centra.

In *C*_vol(max)_ the dorsal ribs were flared laterally to an extreme extent such that the tuberculum still made contact with the diapophysis but the capitulum no longer articulated with the parapophysis. This created a transversely very broad abdominal region. In contrast, in the *C*_vol(min)_ model the ribs were swung much closer to the midline such that the capitulum maintained contact with the parapophysis but the tuberculum disarticulated from the diapophysis, creating a very narrow abdomen. Finally, in *C*_vol(min)_ the left and right scapulae were brought close together leaving minimal space between the anteromedial margins of the coracoids, and the scapulae were angled such that they lay as near to flat as possible against the surface of the ribcage. In *C*_vol(max)_ the scapulae were flared laterally from the ribcage, defining a very broad shoulder girdle with a large space between left and right coracoids.

**Supplementary Material S4: Application of previously published mass prediction equations**

Total convex hull volumes (*C*_vol_) were converted to estimated body mass using the equation for quadrupedal mammals published by Brassey et al. [5]. This equation is based on raw data originally included in the first application of the convex hulling technique to mass estimation by Sellers et al. [6]. In Sellers et al., *C*_vol_ is multiplied by a value for carcass density in order to derive a ‘predicted mass by density’, which is subsequently regressed against literature mass values. This produced a correction factor of x1.2 (i.e. live body mass is estimated to be 20% greater than the body mass predicted by multiplying *C*_vol_ by carcass density). However this does require a value for density to be assigned from the literature, which in itself is problematic given the variability in reported density values both between species and with different methodologies [3, Supplementary Table 1]. In Brassey et al., *C*_vol_ is directly regressed against body mass thus negating the requirement to select an appropriate modern carcass density to a fossil species. This does however implicitly assume that the density of the fossil individual will fall within the range of densities held by the modern calibration. In the case of *Stegosaurus* and modern mammals this assumption can be upheld given the lack of evidence for postcranial skeletal pneumaticity in ornithischian dinosaurs [7].

Convex hull-based estimates of body mass were compared against mass estimates derived solely from proximal limb bone circumference. Femur circumference was taken as the average value of left and right elements (L = 332 mm and R = 346 mm, average = 339 mm). Only the right humerus is preserved (R = 282 mm circumference). We used the R package ‘MASSTIMATE’ to calculate the average and 95% prediction error estimates of body mass from Campione and Evans’ [8] quadrupedal bivariate and multivariate equations. We used their ‘raw’ equations (as opposed to phylogenetically-corrected equation), as the performance of phylogenetically-corrected regression when predicting body mass of a species not included in the original model is uncertain [9].

As we required values for the 95% prediction intervals of the Anderson et al. [10] and Mazzetta et al. [11] equation, we extracted the raw data from the original publications and re-ran the regression analysis in R. Using the values published in Table III of Anderson et al. [10], we were unable to replicate the values for the intercept given in their equations 1a-1b. Rather, we derived the equation:

log_10_mass = -1.075+2.733log_10_*C*_h+f_

using a type-I OLS regression, upon which we based our subsequent mass estimates. Likewise, using the raw data from Mazzetta et al. [11] we derived the equation:

log_10_mass = -3.415+2.682log_10_*C*_f_

using type-I OLS regression. We apply this equation (as opposed to the original type-II regression published) as we consider type-I regression to be more appropriate in the context of mass prediction.

**Supplementary Material S5: Developmental mass extrapolation (DME)**

It is a well-documented phenomenon that the bodily proportions of juveniles are different from those of adults: i.e. the juveniles of a species are not simply isometrically scaled down versions of adults. Given that ontogenetic scaling departs from interspecific scaling, the application of predictive equations derived from an adult dataset to a subadult is problematic. Developmental mass extrapolation (DME) attempts to correct for this. In DME, the body mass of a ‘known’ adult is estimated using the predictive equation in question, and the adult body mass is subsequently scaled down isometrically on the basis of femoral length [12] to the subadult individual. Here we apply DME to the ‘young adult’ NHMUK R36730 using two larger individuals of *Stegosaurus* considered to be adult.

YPM1853 is by far the largest *Stegosaurus* ever found and comprises only a partial skeleton. No histological data are available, but given its external morphology and extremely large size we can be confident in assuming this is an adult specimen. Femoral length, and femoral and humeral circumference were sourced from Benson et al [13].

YPM1853 femur length = 1348mm

femur circumference = 425mm

humeral circumference = 352mm

estimated mass based on Campion and Evans bivariate quadrupedal equation = 6947 kg

YPM1856 is smaller than YPM1853, yet was histologically classified as an ‘old adult’ (growth stage 4) by Hayashi et al. [2] on the basis of possessing an external fundamental system (suggesting that growth had terminated). Femoral length, and femoral and humeral diameters were sourced from Maidment et al. [14] Midshaft diameters were converted to circumferences using the methodology and data of Benson et al. [13].

YPM1856 femur length = 950mm

femur anteroposterior diameter = 52mm

femur mediolateral diameter = 115mm

estimated femur circumference = 287mm

humeral anteroposterior diameter = 71mm

humeral mediolateral diameter = 94mm

estimated humeral circumference = 352mm

estimated mass based on Campion and Evans bivariate quadrupedal equation = 2879 kg

Body mass for NHMUK R36730 was calculated using DME by multiplying the mass estimates for the YPM specimens calculated above by the cube of the ratio of NHMUK femur length/YPM femur length. The femur length of NHMUK R36730 was taken as the average of left and right (863mm).

YPM1853-based mass estimate = 6947*((863/1348)^3) = 1823 kg

YPM1856-based mass estimate = 2879*((863/950)^3) = 2158 kg

**Supplementary Table S1. Volume of dermal armour of *Stegosaurus* (NHMUK R36730) and subsequent mass estimate**

| Plate Number | Volume (m^3^) |
| --- | --- |
| 1 | 0.000023 |
| 2 | 0.000031 |
| 3 | 0.000035 |
| 4 | 0.000036 |
| 5 | 0.000209 |
| 6 | 0.000168 |
| 7 | 0.000291 |
| 8 | 0.000623 |
| 9 | 0.001120 |
| 10 | 0.001914 |
| 11 | 0.001374 |
| 12 | 0.001801 |
| 13 | 0.003087 |
| 14 | 0.002467 |
| 15 | 0.001515 |
| 16 | 0.001428 |
| 17 | 0.000441 |
| 18 | 0.000287 |
| 19 | 0.000057 |
| Total | 0.016907 |

Following a recent study of ornithischian dermal armour [15], total plate mass is estimated as 33.8 kg assuming the plates were composed of compact bone with a density of 2000 kg/m^3^. This value is considerably lower than the 256 kg estimated for *Stegosaurus* by Maidment et al. [15]. Computed tomography undertaken as part of a separate study has indicated the plates of NHMUK R36730 have undergone considerable taphonomic crushing. Furthermore, the plates were almost certainly covered in a layer of keratin of unknown thickness in life. In this study we use the 33.8 kg mass estimate, as it requires no assumptions regarding retrodeformation or reconstruction of unmineralised tissues. This is therefore likely to reflect a minimum mass for dermal armour. However, given the extent of the divergence between volumetric and linear mass predictions of *Stegosaurus* presented here, a mass for dermal armour ranging between 33–256 kg does not significantly affect our interpretation.

**Supplementary Table S2.** Previously published volumetric mass estimates for stegosaurs. **CV**, Chongqing Natural History Museum, P. R. China; **MB**, Museum für Naturkunde, Berlin, Germany; **USNM**, National Museum of Natural History, Smithsonian Institution, Washington, D.C., U.S.A., **ZDM**, Zigong Dinosaur Museum, Sichuan, P.R. China. *converted from volume assuming a density of 1000kg/m^3^.

| **Species** | **Accession No.** | **Mass (kg)** | **Source** | **Comments** |
| --- | --- | --- | --- | --- |
| *Stegosaurus* sp. | NA | 1780 | Colbert 1962 [16] | Sculpted scale model. Not clear which specimen the model is based upon |
|  | NA | 3100 | Alexander 1985 [17] | As above |
| *Stegosaurus stenops* | USNM 4934 | 2200 | Paul [18] | Sculpted scale model method. |
|  |  | 2530 | Henderson [19] | 3D mathematical slicing method |
|  |  | 2610 | Seebacher [20] | Polynomial equation method |
|  |  | 2704 | Maidment et al. [15] | 3D mathematical slicing method |
| *Tuojiangosaurus multispinus* | CV209 | 2750 | Paul [18] | Sculpted scale model method. |
|  |  | 1134 | Seebacher [20] | Polynomial equation method |
| *Kentrosaurus aethiopicus* | MB composite | 321 | Seebacher [20] | Polynomial equation method |
|  |  | 1073-1268* | Mallison [21] | Volumetric CAD sculpting |
| *Huayangosaurus taibai* | ZDM T7001 | 301 | Seebacher [20] | Polynomial equation method |

**Supplementary Table S3.** Previously published mass estimates based on limb dimensions for stegosaurs. Abbreviations as above and **NHMUK**, The Natural History Museum, London, U.K., **SMA**, Sauriermuseum, Aathal, Switzerland, **YPM**, Peabody Museum, Yale University, Connecticut, U.S.A. * now catalogued as NHMUK R36730.

| **Species** | **Accession No.** | **Mass (kg)** | **Source** | **Comments** |
| --- | --- | --- | --- | --- |
| *Stegosaurus stenops* | SMA RCR0603* | 3800 | Redelstorff and Sander [1] | Based on Anderson’s [10] equation modified by Alexander [22] |
| *Stegosaurus mjosi* | SMA VF01 | 2300 | Redelstorff and Sander [1] | Based on Anderson’s [10] equation modified by Alexander [22] |
|  | SMA 0092 | 2500 | Redelstorff and Sander [1] | As above |
|  | SMA 0018 | 4300 | Redelstorff and Sander [1] |  |
|  |  | 4950 | Campione and Evans [8] | Based on femoral and humeral circumference |
|  |  | 4722 | Benson et al. [13] | Applying the equation of Campione and Evans [8] |
| *Stegosaurus stenops* | YPM 1853 | 6954 | Benson et al. [13] | As above |
| *Loricatosaurus priscus* | NHMUK R3167 | 2840 | Benson et al. [13] |  |
| *Gigantspinosaurus sichuanensis* | ZDM 0019 | 2221 | Benson et al. [13] |  |
| *Kentrosaurus aethiopicus* | MB composite | 1597 | Benson et al. [13] |  |

**Supplementary Table S4.** Comparison of linear dimensions between stegosaur specimens USNM 4934 and NHMUK R36730.

| **Element** | **Measurement** | **USNM 4934 (mm)** | **NHMUK R36730 (mm)** |
| --- | --- | --- | --- |
| Dorsal vertebra 8 | Centrum length | 98 | 92 |
|  | Anterior articular facet height | 112 | 80 |
|  | Anterior articular facet width | 70 | 92 |
| Right humerus | Length | 530 | 450 |
|  | Transverse width, distal end | 250 | 160 |
|  | Transverse width, proximal end | 325 | 305 |
| Right ulna | Length | 530 | 412 |
| Right radius | Length | 410 | 312 |
| Right ilium | Preacetabular process length | 530 | 465 |
| Right pubis | Prepubis length | 445 | 276 |
| Right femur | Length | 1060 | 868 |
| Left femur | Length | 990 | 857 |
| Right tibia | Length including fused tarsals | 650 | 498 |

**References**

1. Redelstorff, R. & Sander, P. M. 2009 Long and girdle bone histology of *Stegosaurus*: implications for growth and life history. *J. Vertebr. Paleontol.* **29**, 1087–1099. (doi:10.1671/039.029.0420)

2. Hayashi, S., Carpenter, K. & Suzuki, D. 2009 Different growth patterns between the skeleton and osteoderms of *Stegosaurus* (Ornithischia: Thyreophora). *J. Vertebr. Paleontol.* **29**, 123–131. (doi:10.1080/02724634.2009.10010366)

3. Wu, C. 2011 VisualSFM: A visual structure from motion system.

4. Cignoni, P., Corsini, M. & Ranzuglia, G. 2008 Meshlab: an open-source 3d mesh processing system. *Ercim News* **73**, 45–46.

5. Brassey, C. A. & Sellers, W. I. 2014 Scaling of convex hull volume to body mass in modern primates, non-primate mammals and birds. *PLoS One* **9**, e91691. (doi:10.1371/journal.pone.0091691)

6. Sellers, W. I., Hepworth-Bell, J., Falkingham, P. L., Bates, K. T., Brassey, C. A., Egerton, V. M. & Manning, P. L. 2012 Minimum convex hull mass estimations of complete mounted skeletons. *Biol. Lett.* **8**, 842–845.

7. Butler, R. J., Barrett, P. M. & Gower, D. J. 2012 Reassessment of the evidence for postcranial skeletal pneumaticity in Triassic archosaurs, and the early evolution of the avian respiratory system. *PLoS One* **7**, e34094. (doi:10.1371/journal.pone.0034094)

8. Campione, N. E. & Evans, D. C. 2012 A universal scaling relationship between body mass and proximal limb bone dimensions in quadrupedal terrestrial tetrapods. *BMC Biol.* **10**, 60.

9. De Esteban-Trivigno, S. & Köhler, M. 2011 New equations for body mass estimation in bovids: Testing some procedures when constructing regression functions. *Mamm. Biol. - Zeitschrift für Säugetierkd.* **76**, 755–761. (doi:10.1016/j.mambio.2011.07.004)

10. Anderson, J. F., Hall-Martin, A. & Russell, D. A. 1985 Long-bone circumference and weight in mammals, birds and dinosaurs. *J. Zool.* **207**, 53–61.

11. Mazzetta, G., Christiansen, P. & Farina, R. 2004 Giants and Bizarres: Body size of some Southern South American Cretaceous Dinosaurs. *Hist. Biol.* , 1–13.

12. Erickson, G. M. & Tumanova, T. A. 2000 Growth curve of *Psittacosaurus mongoliensis* Osborn (Ceratopsia: Psittacosauridae) inferred from long bone histology. *Zool. J. Linn. Soc.* **130**, 551–566.

13. Benson, R. B. J., Campione, N. E., Carrano, M. T., Mannion, P. D., Sullivan, C., Upchurch, P. & Evans, D. C. 2014 Rates of dinosaur body mass evolution indicate 170 million years of sustained ecological innovation on the avian stem lineage. *PLoS Biol.* **12**, e1001853. (doi:10.1371/journal.pbio.1001853)

14. Maidment, S. C. R., Linton, D. H., Upchurch, P. & Barrett, P. M. 2012 Limb-bone scaling indicates diverse stance and gait in quadrupedal ornithischian dinosaurs. *PLoS One* **7,** e36904. (doi:10.1371/journal.pone.0036904)

15. Maidment, S. C. R., Henderson, D. M. & Barrett, P. M. 2014 What drove reversions to quadrupedality in ornithischian dinosaurs? Testing hypotheses using centre of mass modelling. *Naturwissenschaften* **101**, 989-1001 (doi:10.1007/s00114-014-1239-2)

16. Colbert, E. 1962 The weights of dinosaurs. *Am. Museum Novit* **2076**, 1-16.

17. Alexander, R. M. N. 1985 Mechanics of posture and gait of some large dinosaurs. *Zool. J. Linn. Soc.* **83**, 1–25.

18. Paul, G. 1997 Dinosaur models: The good, the bad, and using them to estimate the mass of dinosaurs. In Wolberg, D.L., Stump, E. & Rosenberg, G.D. (eds.) *Dinofest International: Proceedings of a Symposium Sponsored by Arizona State University*. Academy of Natural Sciences, Phlidelphia. 129–142.

19. Henderson, D. M. 1999 Estimating the masses and centers of mass of extinct animals by 3-D mathematical slicing. *Paleobiology* **25**, 88–106.

20. Seebacher, F. 2001 A new method to calculate allometric length-mass relationships of dinosaurs. *J. Vertebr. Paleontol.* **21**, 51–60.

21. Mallison, H. 2011 Defense capabilities of *Kentrosaurus aethiopicus* Hennig , 1915. *Palaeontol. Electron.* **14**, 14.2.10A.

22. Alexander, R. M. N. 1989 *Dynamics of Dinosaurs and Other Extinct Giants*. New York: Columbia University Press.
